# Dissertation sur la meilleure forme des souliers
*The following mottoes are prefixed to the work."Calceus pede major subvertit, minor urit.Horat.""Non multum absuit, quin sutrinum quoque inven-"tum à sapientibus diceret Posidonius.Seneca."


**Published:** 1783

**Authors:** 


II. *Diſſertation ſur la meilleure forme des ſouliers*. Par M.* Petrus Camper. 8vo. 82 pages, with a copper-plate.

THIS eſſay, of which only a few copies are printed for the author's friends, is a proof that in the hands of a man of genius almoſt

* The following mottoes are prefixed to the work.

" Calceus pede major ſubvertit, minor urit.

HORAT."

" Non multum abfuit, quin ſutrinum quoque inven-

" tum à ſapientibus diceret Poſidonius.

SENECA."

any any topic may be rendered interefting. In his preface, the learned profeffor informs us, that his work originated from a motive of pleafantry. He was defirous, it feems, of convincing fome of his quondam pupils who were maintaining that the fubjects for differtations were exhaufted, that any thing, though feemingly of the moft trifling nature, even a fhoe, might be made of importance by inveftigating it thoroughly, and on philofophical principles. To make good his affertion, he undertook to write a treatife on the beft fhape of fhoes, "on me fit un defi— fays he—" on crut du moins que je n'oferois "jamais le publier fous mon nom. Je me "prêtai a la plaifanterie et j'ecrivis."——Pleafantry apart, however, he wifhes to have it underftood that his arguments on this fubject are founded on attentive obfervation and repeated experiments. He thinks his remarks, if properly attended to, may be of great ufe to mankind; and obferves, that if he attains his view in this refpect, we may fay of him not *ridendo caftigat mores*, but *ridendo calceos corrigit*.

In his Introduction to the work the author complains of the inattention of former writers to the fubject, and points out its utility and importance. He contends that in this article of of our drefs no improvements have been made for many centuries, the fhoes now in ufe being in no refpect fuperior to thofe of the ancients, the ill effects of which are fo well defcribed by Celfus, Paulus Ægineta, and Aetius.

He had not long reflected on this fubject before it occurred to him that a fhoe fit for one town may be very improper for another; that, for inftance, a fhoe adapted to the level walks of the Hague, will be lefs fuited to Amfterdam, and will be even defective at Groninguen and other places where the ftreets are paved with flints. He obferves alfo, that the common method of meafuring the foot for a pair of fhoes is ill founded, becaufe as the foot lengthens itfelf in walking and becomes fhorter when inactive, a fhoe made by meafuring the foot in the latter ftate muft neceffarily be too fhort, and of courfe will comprefs the heel and great toe. He is alfo convinced that the heel of the fhoe fhould be brought more forward under the fole of the foot than it ufually is, in order to fupport the centre of gravity; and that it fhould be made higher for an irregular pavement than when we walk on plain ground.

As proofs of the variety requifite in the form of fhoes, he obferves, that all perfons do not walk alike. Thus women walk differently from men, becaufe their haunches are wider; and in the advanced ftate of pregnancy the upper part of their body inclines backwards to fupport the centre of gravity, fo that under thefe circumftances the greater number of women walk on their heels. Again, children differ from adults in their manner of walking on account of the fhortnefs of their legs; while old perfons, from having their head and whole body inclined too much forwards, are obliged to bend their knees to fupport the centre of gravity, which neceffarily falls more on the back of the foot.

The work itfelf is divided into feven chapters. In the firft, fecond, and third of thefe, the author defcribes the mechanifm of the foot, which he confiders with great precifion both as an anatomift and a mathematician. He proves from the ftructure of its bones, and the arrangement of its mufcles, that it might be capable of many ufes, were not its fhape deformed by the fhoes in common ufe. The great toe, he obferves, is fhorter than that next to it, though not fo much fo as it is reprefented in ancient ftatues, and in the pictures and engravings of Van Haerlem, Goltzius, and other artifts of the fixth cencentury.—In women who wear ſhoes with very high heels, the head of the aſtragalus, we are told, is bent downwards, and the os naviculare and the oſſa cuneiformia are gradually preſſed inwards. The foot then becomes ſo diſtorted, that women who have been long accuſtomed to ſhoes of this kind cannot walk in flat ſlippers or bare-footed without feeling pain in the calf of the leg, becauſe the muſcles which compoſe that part and unite to form the tendo Achillis are no longer ſuſceptible of much tenſion. Our profeſſor likewiſe confirms the truth of an obſervation made by Andry (*Traité ſur l'Orthopedie*, tome I. p. 68.) that high heel ſhoes are liable to occaſion a curvature of the ſpine; and ſeveral ingenious remarks are added, to prove that the ſame evil frequently operates as a cauſe of narrow pelvis and difficult parturition.

In the fourth chapter the author gives a theory of walking, founded on his own obſervations and thoſe of Borelli. In the fifth he treats of the properties of the ſhoe (*les proprietés du ſoulier*) and in the following chapter he deſcribes what he thinks is the beſt ſhape for this purpoſe. The reſults of his obſervations on this, which may be deemed the practical part of his ſubject, are, 1. That the length of the ſole of the ſhoe ſhould be proportioned to the length of the foot in walking, and of courſe that the ſhoemaker ought to meaſure the foot in a flat, and afterwards in a bent ſtate. 2. That each foot requires a ſhoe of a different ſhape. This, we are told, is agreeable to what is practiſed by the peaſants in Holland. 3. That the exact width of the foot ought to be meaſured with a pair of compaſſes. 4. That the toe of the ſhoe ought to be round, and a little elevated to enable us to paſs over an irregular ſurface with greater eaſe. 5. That the height of the heel ought to be proportioned to the inequality of the pavement, and brought ſufficiently forward under the heel of the foot to receive or rather ſupport the centre of gravity. 6. That the upper-leather and quarters ſhould be ſo contrived, that the buckle ſhall touch the oſſa cuneiformia preciſely at that part where they are connected with the metatarſal bones of the great toe, and of the two others next to it.

In the ſeventh chapter we have an account of the diſeaſes, ſuch as corns, warts, &c. occaſioned by bad ſhoes, and the means of curing them. Here, as in every other part of the treatiſe, we meet with many ingenious and uſeful ful obfervations which do not admit of abridgment, and for which we muft refer our readers to the work itfelf.

